# Analysis of the effect of motion on highly accelerated 3D FatNavs in 3D brain images acquired at 3T

**DOI:** 10.1371/journal.pone.0306078

**Published:** 2024-07-25

**Authors:** Elisa Marchetto, Daniel Gallichan

**Affiliations:** 1 CUBRIC/School of Engineering, Cardiff University, Cardiff, United Kingdom; 2 Center for Biomedical Imaging, Department of Radiology, New York University Grossman School of Medicine, New York, New York, United States of America; 3 Center for Advanced Imaging Innovation and Research (CAI2R), Department of Radiology, New York University Grossman School of Medicine, New York, New York, United States of America; Museo Storico della Fisica e Centro Studi e Ricerche Enrico Fermi, ITALY

## Abstract

**Purpose:**

3D FatNavs are rapid acquisitions of MRI fat-volumes within the head that can be used for retrospective motion correction for brain MRI. 3D FatNavs typically use very high acceleration factors and are reconstructed with the GRAPPA parallel imaging technique. However, the GRAPPA reconstruction is not expected to perform well on 3D FatNavs volumes in the presence of strong motion due to the mismatched calibration data acquired once at the start of the scan, leading to motion-parameter misestimation. This study aims to assess the accuracy and precision of 3D FatNav-derived motion-estimates in the presence of large changes in head position.

**Methods:**

Rigid motion parameters were simulated and applied retrospectively to the 3D FatNav volumes from MPRAGE datasets acquired at 3T. The transformed images were then re-reconstructed using GRAPPA to simulate real motion deterioration of the fat-navigator, and used to estimate the motion applied and evaluate the tracking inaccuracy. This information was then used to estimate the residual motion after 3D FatNav-based motion correction and applied to the original MPRAGE volumes. The effect of the misestimation was assessed using an image quality metric and the evaluation scores from two observers. Quality boundaries were then estimated to assess the motion tolerance when 3D FatNavs are used.

**Results:**

The GRAPPA reconstruction was shown to deteriorate for large changes in the head position, affecting the quality of 3D FatNav volumes and consequently degrading the accuracy of the motion-estimates. Based on our simulations, the estimated threshold of motion that led to a noticeable degradation in the MPRAGE image quality was up to RMS values of 3.7° and 3 mm for rotations and translations respectively.

**Conclusions:**

3D FatNavs were shown to be able to correct for a wide range of motion levels and types. Boundaries of acceptable motion magnitudes for different levels of acceptable loss of image quality were determined.

## 1. Introduction

A retrospective motion correction technique for brain MR images has been proposed by Gallichan et al. [1] to detect and correct non-deliberate motion during high-resolution imaging. The idea consists of applying a 3D GRE sequence combined with a three-pulse fat-selective binomial excitation as a navigator. Because of the natural sparsity of fat images, it is possible to apply the GRAPPA [2] parallel imaging technique to acquire exceptionally highly accelerated fat-volumes as navigators (*3D FatNavs*): in this way small motion can be detected and corrected. The acceleration factor (R) defines the amount of k-space data collected during the 3D FatNav acquisition: the higher the acceleration factor, the faster the acquisition would be. An acceleration of 4x4 (corresponding to R = 16) denotes that one sample in k-space is acquired every 4 lines in the phase and partition encoding directions. To reconstruct the missing lines, GRAPPA uses the auto-calibration signal (ACS) lines, which constitute the fully-sampled central region of a 3D FatNav volume collected prior to or during the main acquisition, with only a few seconds added to the scan total duration. Motion correction based on 3D FatNavs has been used to improve the image quality in MR images of the brain affected by non-deliberate motion [1, 3] and by deliberate motion [4, 5]. In [1], image sharpness was restored in high-resolution images by including the accelerated 3D FatNavs as part of an MP2RAGE and a TSE protocol with negligible increase in scanning time. The use of navigator-based methods such as 3D FatNavs has the advantage that no extra hardware is required, making it more convenient to use compared to marker-based tracking methods such as the Moiré Phase Tracking (MPT) device (Metria Innovation, Milwaukee, WI). Unlike 3D FatNavs, MPT requires the use of a marker fixed on a mouthpiece extension, which is held in position by the subject’s upper jaw, while a single camera acquires 86 frames/s. The two methods have been shown to give comparable results in the case of deliberate and non-deliberate motion [6]. Moreover, both techniques could completely compensate for artifacts caused by slow motion, which may occur in more compliant subjects [6].

3D FatNavs have been tested in 40 patients with diagnosed or suspected brain tumors on a clinical 3T scanner (MAGNETOM Skyra 3T, Siemens Healthcare, Erlangen, Germany), resulting in visible improvements in image quality after motion correction [3], demonstrating to be a valuable tool for motion correction in clinical brain MRI. Moreover, its negligible additional scanning time and no need for extra hardware makes it likely to be preferable as a motion correction solution for less compliant subjects who might not tolerate long scans or needing to wear physical markers on their heads. However, GRAPPA reconstruction is not expected to perform well on 3D FatNav volumes in the presence of strong head position changes. This is due to the mismatched calibration data acquired once at the start of the scan and the data for each individual 3D FatNav volume being reconstructed: if the head position has changed substantially, then the GRAPPA calibration may be sub-optimal and affect the quality of the image reconstruction. The compromised GRAPPA reconstruction is then expected to lead to motion-parameter misestimation.

This study aims to assess the accuracy of motion parameters derived from 3D FatNavs in the presence of large changes in the head position, analyzing the relationship between the extent of the motion and the expected degradation in the 3D FatNav volumes and the quality of the image corrected using the position estimates generated using these 3D FatNav volumes. This was performed by simulating a broad range of motion scenarios on 3D FatNav volumes acquired with and without deliberate motion to analyze the effect on the motion estimation accuracy in relation to the GRAPPA reconstruction. The results were used to derive empirical limits of the motion that the current implementation of the 3D FatNavs technique can compensate to produce an artifact-free image.

## 2. Methods

### 2.1 Data acquisition

Five different datasets of MPRAGE brain MR images were acquired on a Prisma scanner (Siemens Healthcare, Erlangen, Germany) at 1 mm isotropic resolution, with TI/TE/TR = 1100/3.03/2410 ms, FA = 8° and R = 2, using a 64-channel head coil with a total scanning time of 5:38 min. Two of the five datasets were acquired with deliberate motion, while the other three datasets were acquired without. Interleaved with the MPRAGE sequence, and following each readout train, a 3D FatNav volume at 4 mm isotropic resolution was acquired as navigator, with TE/TR = 1.43/3.4 ms and acceleration factor R = 16 (ACS data matrix size 20x32, GRAPPA reconstruction kernel size 2x2), resulting in a total acquisition time of 0.31 s for each 3D FatNav volume.

Ethical approval for this study was obtained from Cardiff University School of Psychology Ethics Committee board. The five healthy participants were recruited to take part in the study between the 19^th^ of October 2020 and the 31^st^ of March 2021. Written informed consent was obtained from all subjects before the study.

### 2.2 B_1_ maps

**Fig 1:**
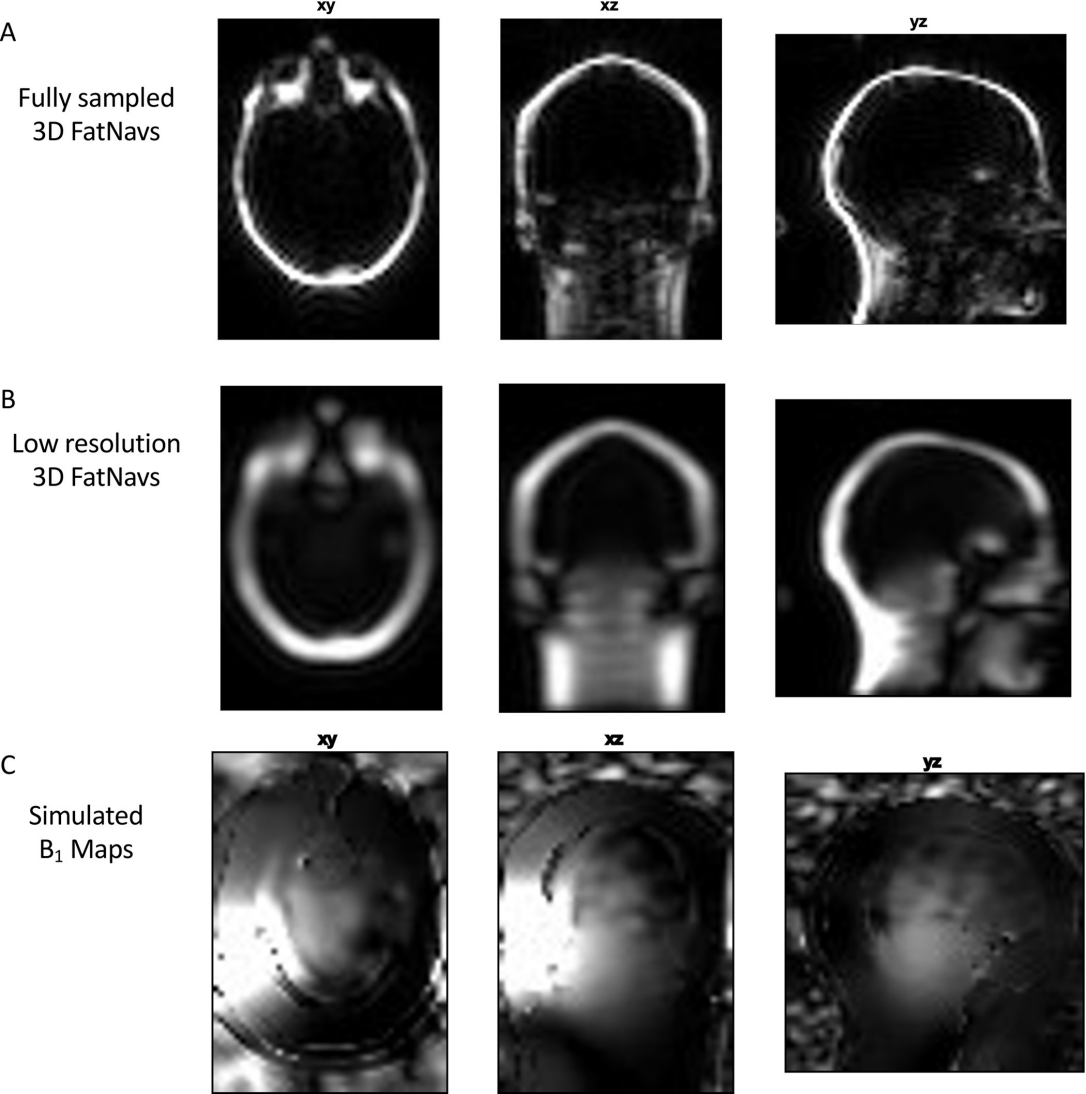
B_1_ maps estimation. (A) Original 3D FatNav volume acquired at 4 mm isotropic resolution. (B) A low-resolution 3D FatNav volume was obtained from the ACS lines obtained from the original data via the iFFT. (C) To calculate the B_1_ maps, the low-resolution 3D FatNav volume was divided by the root-sum-of-squares of all channels’ coil volumes. (C) The B_1_ field maps of one channel calculated for one dataset. Maps were calculated for each channel of all the five datasets using the correspondent ACS lines acquired prior to the scan. Notice how the field is smooth close to the fat layer, but the SNR degrades rapidly towards the centre of the brain.

The 3D-FatNav ACS data from the pre-scan was filtered using an anti-aliasing Tukey window and extended to the entire FOV, using the zero-filling method. A low-resolution 3D FatNav volume was generated by applying the iFFT to the filtered ACS data and then processed using a 3D Hamming window to reduce ringing. B_1_ maps were calculated as the ratio between each coil channel’s pre-reconstructed 3D FatNav volume and the root-sum-of-squares of all channels’ volumes. The maps were then smoothed using an spline smoother for data of arbitrary dimension (*smoothn* function [7]) to ensure overall smoothness of the coil sensitivity maps. Fig 1 summarizes all the steps performed to estimate the B_1_ maps: the use of fat images boosted the smoothness of the B_1_ maps in the fat region around the skull, but the SNR degrades rapidly towards the centre of the brain. Despite not providing a perfect representation of the B_1_ profile, the use of fat images is still preferred over water images (e.g. the available MPRAGE images themselves), which will be affected by discontinuities in phase in the region around the fat layer, where the dominant signal in the image flips from water to fat.

### 2.3 Tracking accuracy from 3D FatNavs

A high-resolution 3D FatNav volume was reconstructed using the GRAPPA parallel imaging technique for each dataset (Fig 1A). Different combinations of simulated motion parameters were then applied to the high-resolution 3D FatNav volumes in the image domain, using the SPM realign tool [8]. Rotational and translational motion parameters were generated by separately selecting rotations ranging between 0 to 20° with increments of 5° (125 combinations), while translations were selected from a range up to 40 mm with 10 mm steps (125 combinations). Displacement along the z-direction was also applied in the negative sense (corresponding to movement towards the feet) as when a head is imaged in-situ the achievable motion along the superior/inferior direction is strongly restricted due to the presence of the head coil (100 combinations). Translations and rotations were considered symmetrical in all other dimensions.

**Fig 2:**
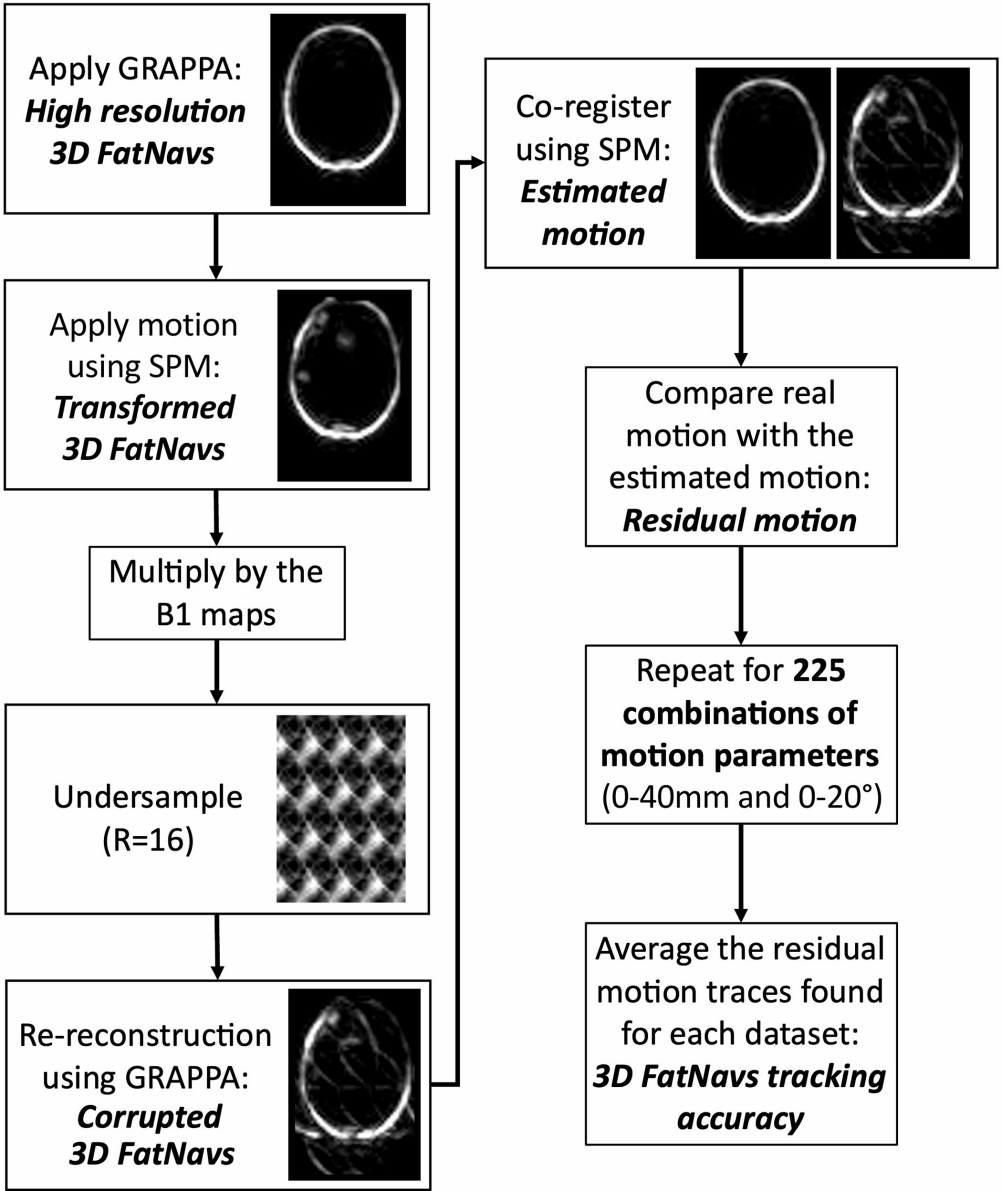
Schematic representation of the process performed to calculate the 3D FatNavs tracking accuracy for 225 different combinations of motion parameters (ranged between 0–40 mm translations and 0–20° rotations). One set of 6 motion parameters were applied to the high-resolution 3D FatNav volumes (obtained via GRAPPA reconstruction) using SPM. The transformed 3D FatNav volumes were multiplied by the B_1_ maps to find the transformed volume for each coil. Each volume was then undersampled using an acceleration factor of R = 16 (4x4) and re-reconstructed using GRAPPA to simulate the final corrupted 3D FatNav volumes if head motion occurred during the scan. By co-registering the initial high-resolution volume and the corrupted volume it was possible to find the estimated motion parameters if 3D FatNavs were used. These motion parameters were compared to the real motion parameters applied to find the residual motion (or estimation error). The same steps were repeated for all motion traces for each dataset. The residual motion was averaged across the three datasets to find the 3D FatNavs tracking accuracy, from which it was possible to estimate the residual motion from any new motion trace via linear interpolation.

These modified 3D FatNav volumes were multiplied by the B_1_ maps in the complex domain, Fourier transformed and undersampled using an acceleration factor R = 16 to simulate the raw data for each coil channel. Each volume was then re-reconstructed using GRAPPA to simulate the final corrupted 3D FatNav volume. A comparison between the modified and the corrupted 3D FatNav volumes was performed to analyze the extent of the GRAPPA corruption. A total of 225 corrupted 3D FatNav volumes were generated for each acquired dataset by combining the 125 rotational parameters to the 125 and 100 sets of translations. Corrupted and reference 3D FatNav volumes were co-registered using SPM to obtain an estimate of the motion parameters. This was then compared to the real motion applied to find the mis-estimation caused by the GRAPPA inconsistency. The result was averaged between datasets for the full range of parameters considered. The residual motion could be therefore estimated from any given true motion using linear interpolation on the 3D FatNavs tracking accuracy, where the residual motion represented the apparent head motion after 3D FatNavs-based correction. Fig 2 shows a schematic representation of the steps to obtain the corrupted 3D FatNav volumes and the residual motion parameters forming the 3D FatNavs tracking accuracy.

### 2.4 MPRAGE simulations

**Fig 3:**
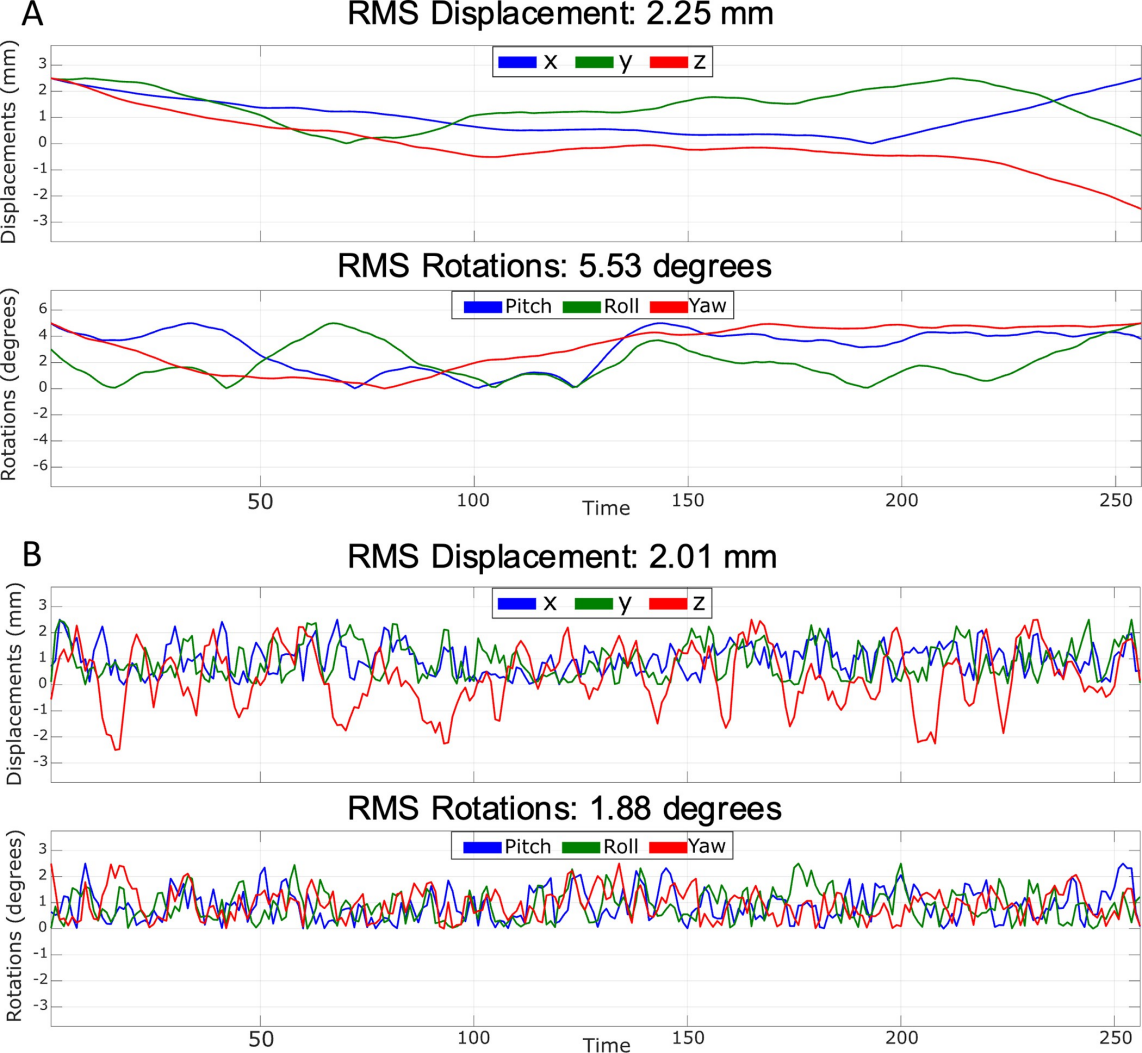
Smooth and rough motion parameters. Example of smooth (A) and rough (B) motion parameters randomly generated with RMS = 2.25 mm and RMS = 5.53° and RMS = 2.01 mm and RMS = 1.88° for translations and rotations.

In this study, different combinations of true motion curves were randomly generated using a fractal noise generator (similar to how Perlin noise [9] is often used in computer graphics applications). The function used is available in the *retroMoCoBox* toolbox [10] and it requires the number of motion parameters to be generated and a weighting factor. The function generates multiple arrays of numbers at different frequencies and amplitudes using a cubic interpolation function and smoothed using the weighting factor. The function then sums all these randomly generated arrays to produce the final fractal noise signal. In this work, we generated two different types of motion, which we have chosen to refer to as smooth and rough motion. Rough motion is characterized by rapid and abrupt changes in the head position, occurring for a prolonged period, while smooth motion is characterized by slow changes in the head position. Examples of motion traces for smooth and rough motion are reported in Fig 3.

96 different combinations of motion parameters were therefore generated for smooth and rough types of motion, with RMS values ranging between 0–20° and 0–40 mm for rotations and translations respectively, generating 192 combinations for each of the three datasets for a total of 576 motion curves.

Motion occurring in the centre of the k-space has more impact on the final image than motion occurring at the edges due to the different types of information encoded in the k-space: the k-space centre contains low frequency information related with the contrast and shape of the image, while edges and details are encoded in the high spatial frequencies’ information at the k-space periphery [11]. To take this into account, the motion parameters were randomly generated four times for each range of motion: the motion distribution was then expected to vary during each repetition, but with little change in the RMS value.

The motion parameters previously applied to the 3D FatNav volumes, with the process shown in Fig 2, were used as sample points and the correspondent residual motion as sample values. Using linear interpolation, it was possible to estimate the expected residual motion (if 3D FatNavs were used) for translations and rotations separately based on the new motion parameters generated using the fractal noise. The corresponding residual motion was applied to the three original MPRAGE images acquired without voluntary motion using the retroMoCoBox toolbox, simulating the predicted residual degradation in image quality that would be expected if 3D FatNavs had been used to correct motion of this type. The original motion parameters generated using the fractal noise where directly applied to the MPRAGE images to simulate the “no motion correction” case.

### 2.5 Image quality assessment

Image quality evaluation was initially performed using the gradient entropy (GE) metric, which was found by McGee et al. as the best metric for autocorrection of MR images of the shoulder, as the metric closely correlated with observers’ evaluations [12]. In this study, the GE metric was calculated by first estimating the gradients along x and y as convolution between the Prewitt operators and the image (function *imfilter)*. The gradient magnitude was then estimated as the square root of the sum-of-squares of the two gradients, from which we derived the entropy of the magnitude gradient (*entropy* function).

**Fig 4:**
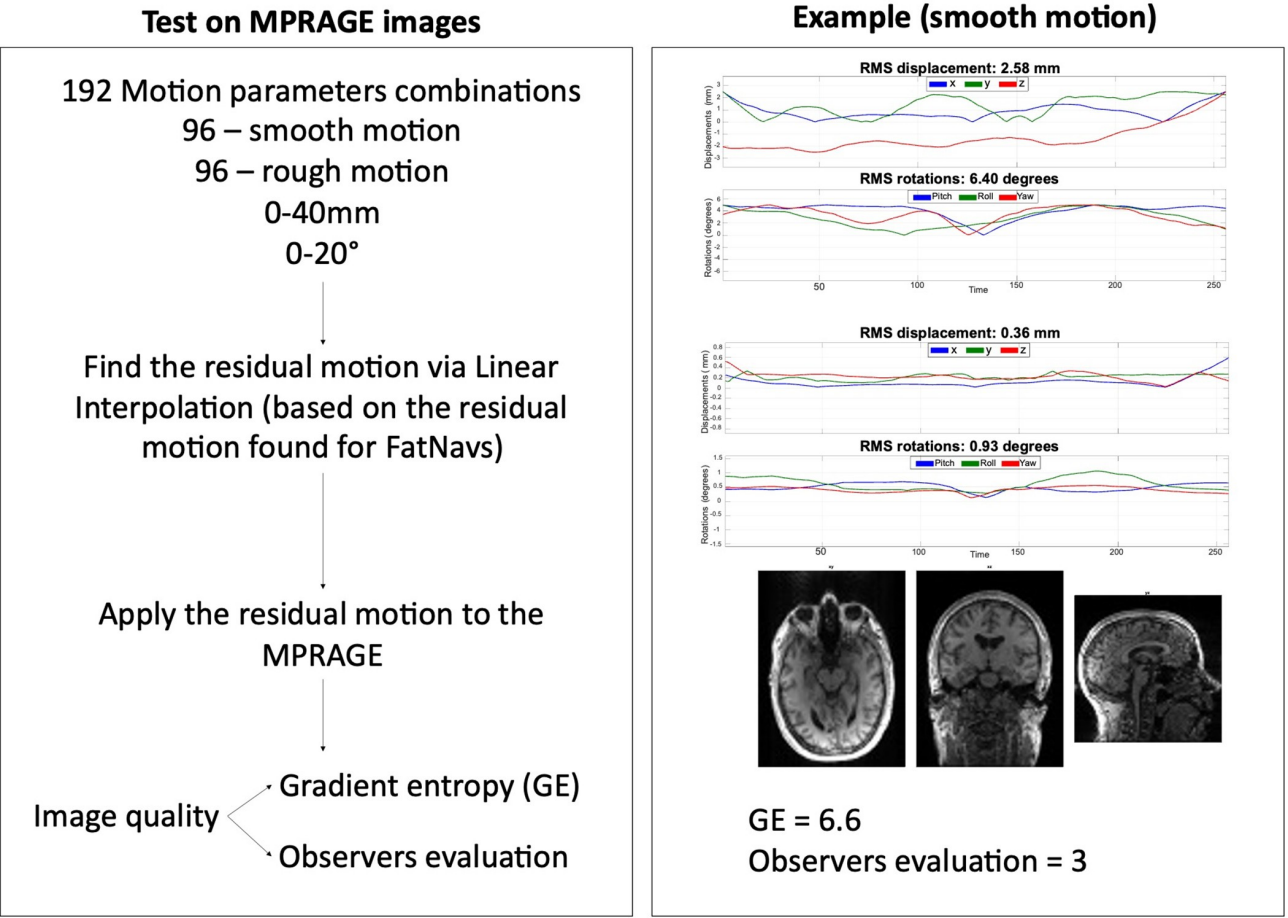
Summary of the process used to test the 3D FatNavs tracking accuracy on MPRAGE images, together with an example for a set of smooth motion parameters. A total of 192 motion traces were generated, 96 for smooth motion and 96 for rough motion, within a range of 0–40 mm translations and 0–20° rotations. For each motion trace, the residual motion was found via linear interpolation of the 3D FatNavs motion accuracy previously found (Fig 2). The residual motion was then applied to the motion-free MPRAGE volume. The image quality assessment was performed using the GE metric and evaluation from an observer using a scale between 1 to 4, with 4 denoting no visible motion artifacts.

Two observers, non-clinicians and with 15- and 3- years’ experience in brain MRI, evaluated the 192 MPRAGE images generated for each of the three datasets, acquired without deliberate motion, using a Graphical User Interface (GUI) created in MATLAB: the image was displayed on the screen with no information regarding the GE value or the other observer’s evaluation; the observer scored each image with a scale from 4 to 1, with 4 = no visible motion artifacts, 3 = some motion artifacts, 2 = strong motion artifacts and 1 = severe motion artifacts. A diagram of this process (together with an example for a set of smooth motion parameters) is reported in Fig 4.

### 2.6 Statistical analysis

The next sections briefly describe the statistical tests performed in this study. A summary is reported in Table 1, together with the MATLAB functions used.

**Table 1:** Summary of the statistical analysis.

Test	Aim	Function (MATLAB)
Inter-observer variability (*Krippendorff’s alpha)*	Measure agreement among observers	*krialpha*.*m*
Multinomial logistic regression	Analyse relation between GE and the observers’ evaluations	*mnrfit*.*m*
One-way ANOVA	Test the model found by the multinomial logistic regression	*anova1*.*m*
Linear regression between RMS and GE	Find image quality category boundaries based on RMS	*fitlm*.*m* *step*.*m*
Linear regression between FD and GE	Find image quality category boundaries based on FD	*fitlm*.*m*
Non-linear regression between FD and GE	Find image quality category boundaries based on FD	*fitnlm*.*m*

#### 2.6.1 Inter-observer variability

The inter-observer variability was measured using the Krippendorff’s Alpha coefficient [13], which is a statistical measure developed to calculate the agreement among observers. The function *kriAlpha* [14] was used in MATLAB to estimate the coefficient.

#### 2.6.2 Multinomial logistic regression

A multinomial logistic regression was performed revealing how the probability of falling in an evaluation category (4 to 1) would change based on the GE values [15]: the probability of falling into categories representing good quality images (namely categories 4 and 3) was expected to be inversely proportional to the GE value. The multinomial logistic regression was performed using the *mnrfit* function, followed by a one-way ANOVA test (function *anova1*).

#### 2.6.3 Linear regression between RMS and GE

The relationship between the motion occurred and the GE was investigated using the linear model shown in the following equation:

$$Y=1+a\cdot X_{1}+b\cdot X_{2}+c\cdot X_{1}\cdot X_{2}$$ (1)

the output variable *Y* represented the GE, while *X*1, *X*2 described the rotational and translation motion with their respective parameters *a* and *b*; an interaction term was also added to the model, to describe the mutual effect that rotations and translations (independent variables) might have on the outcome.

We used a linear regression analysis to find the best coefficients fitting the linear model described in Eq 1 (function *fitlm*). We also tested whether the presence of the interaction term significantly improved our linear model using the Bayesian Information Criterion (BIC) [16], which is a model selection criterion that uses a penalty term based on the number of parameters to avoid overfitting: the model with lowest BIC value was selected as the best fitting model (function *step*). The motion magnitude was calculated for rotational and translational motion separately as the RMS value along each direction and averaged across datasets. The model was used to find the motion threshold between each evaluation’s category. This was performed by selecting the GE values at the intercepts between each category boundary, found via the multinomial logistic regression previously performed. It was possible to estimate the rotational or translational motion corresponding to the entropy value by placing the other to zero. The level of motion corresponding to each category boundary was evaluated twice: once assuming that the simulated motion was the true motion (i.e no 3D FatNav-based correction applied) and once assuming that the simulated motion was the residual motion following 3D FatNav-based correction. This allows a comparison of the expected increase in motion that can be tolerated when 3D FatNav-based correction is used.

#### 2.6.4 Linear and non-linear regression between FD and GE

Although the RMS of rotations and translations provides information regarding the magnitude of the motion occurred, it cannot effectively compare the motion estimation accuracy in the case of smooth vs rough motion, as smooth and rough motion profiles with similar RMS values are expected to have quite a different level of impact on the amount of motion-artifacts in the resulting image.

In this study, the Framewise Displacement (FD) was adopted as a single-value metric to measure the head motion between consecutive volumes. It was calculated as the sum of the absolute values of the derivatives of the six realign parameters, where rotations were estimated as the displacement occurring on the surface of a sphere of 50 mm radius [17]. The relationship between the mean FD (averaged across the entire acquisition) and GE was investigated using a linear and a non-linear regression model (represented in Eq 2, with X and Y being the mean FD and the GE respectively). For the latter, a logarithmic model was chosen due to the entropy being based on the log-function.


Y=a+b∙X

Y=a+b⋅log(X−c) (2)

The information derived from the two models was then used to estimate the FD values that correspond to the boundaries between the 4 coarse categories from the human evaluation of the images using the residual motion following 3D FatNavs motion correction (as shown in Fig 4).

## 3. Results

### 3.1 GRAPPA motion robustness

**Fig 5:**
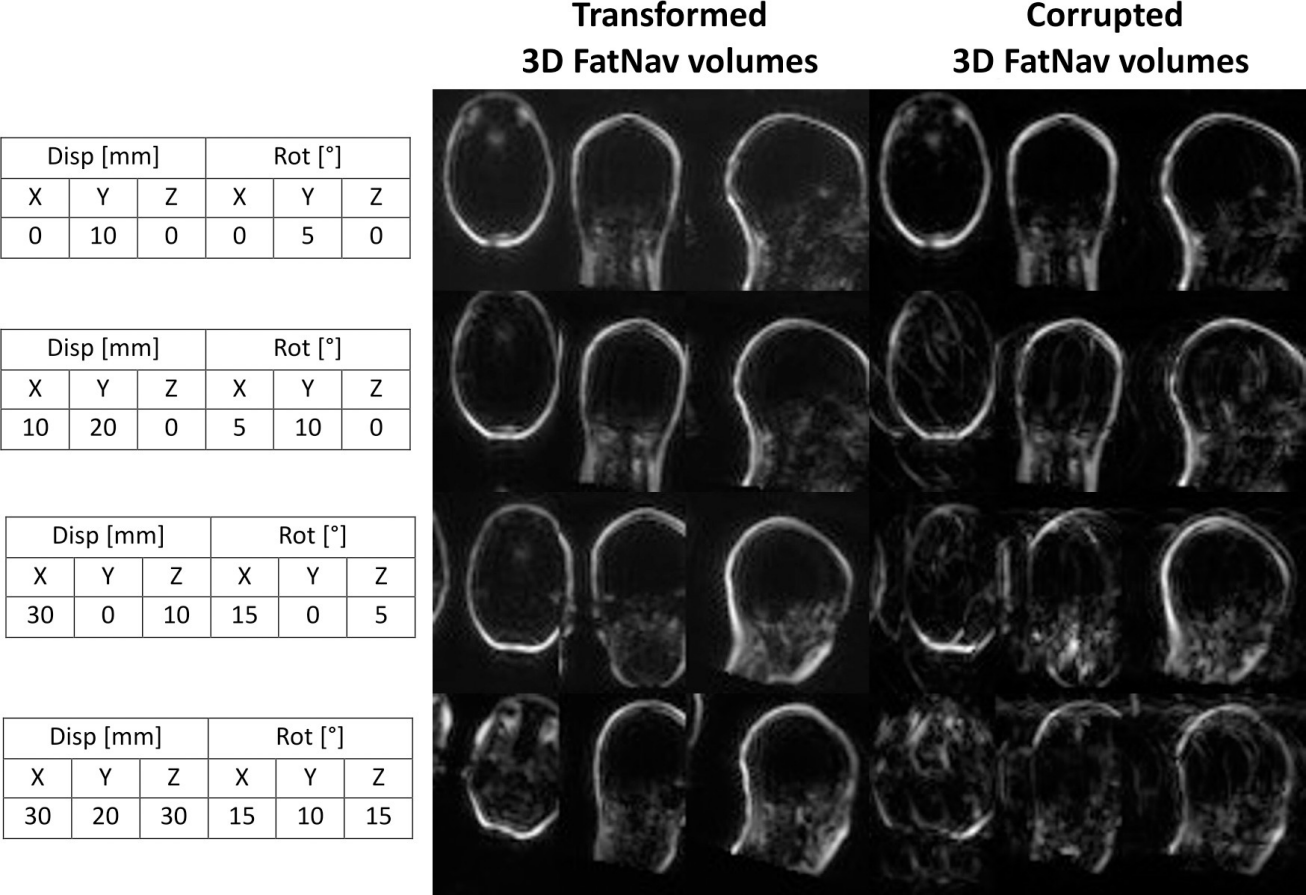
Comparison between 3D FatNavs volume before and after GRAPPA "re-reconstruction". The figure shows the effect of mismatched ACS data to the 3D FatNavs volumes for four different levels of motion (tables on the left): parallel imaging artifacts are shown to increase with the amount of motion. The “Transformed 3D FatNav volumes” are obtained after applying the motion parameters using SPM. The volumes are then multiplied by the B_1_ maps and undersampled using an acceleration factor R = 16. The “Corrupted 3D FatNav volumes” are obtained by reconstructing the undersampled volume.

Fig 5 compares 3D FatNav volumes before and after the GRAPPA "re-reconstruction" step for four different levels of motion (low, medium, high and very high) randomly selected from the 225 different combinations of motion parameters applied to the acquired 3D FatNav volumes. Parallel imaging artifacts were found to increase for larger changes in the head position, due to a more severe mismatch between the ACS lines for the GRAPPA reconstruction and the transformed 3D FatNav volumes.

### 3.2 Inter-observer variability

The Krippendorff’s alpha values calculated between the two observers first evaluations (sample size of 192) were above 0.8 in all three datasets (0.85/0.87/0.90 respectively), denoting high reliability between the two observers [13].

### 3.3 Multinomial logistic regression

**Fig 6:**
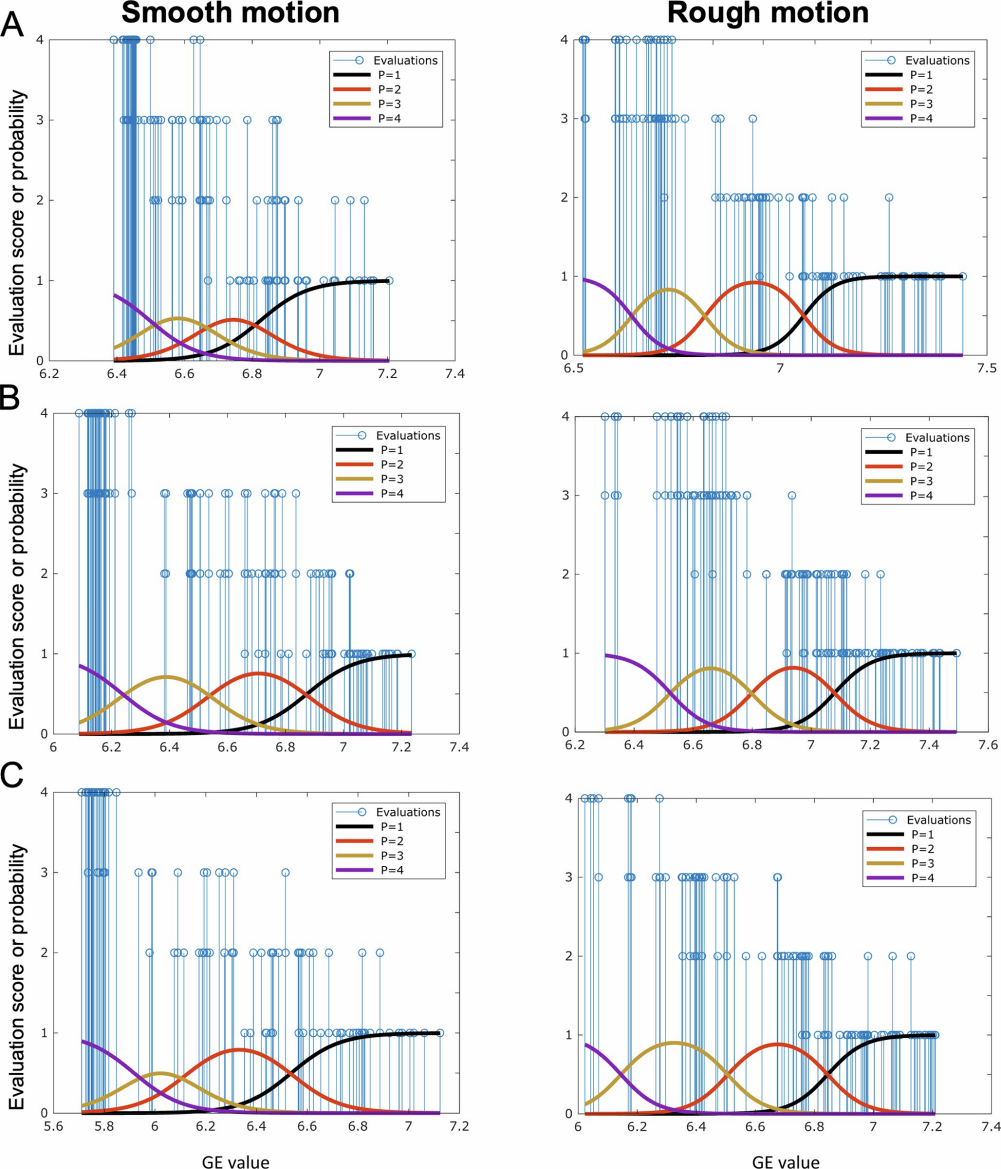
Multinomial logistic regression between image evaluations and the GE in case of smooth and rough motion for dataset (A) 1, (B) 2 and (C) 3. All evaluations, from 1 to 4, are plotted as blue vertical lines, while each colored Gaussian waveform represents the probability of falling in one of the four evaluation’s categories: category 4 (no visible artifacts) in purple, category 3 (some motion artifacts) in yellow, category 2 (strong motion artifacts) in orange and category 1 (severe motion artifacts) in black. Lower entropy values corresponded to higher category rating and, therefore, to a better image quality.

Fig 6 presents the results from the multinomial logistic regression performed between the observers’ evaluations and the image quality relative to each image. Each curve describes the changes in probability distribution based on the GE value among categories. As the GE value increases, the probability of falling into a category representing a low level of motion artifacts decreases, as expected. These results were further corroborated by the one-way ANOVA test, whose results are reported in Table 2.

**Table 2:** One-way ANOVA test.

Dataset	Motion type	F-value (3, 284)	p-value
Dataset 1	Smooth motion	283.06	p<0.001
	Rough motion	200.34	p<0.001
Dataset 2	Smooth motion	563.71	p<0.001
	Rough motion	787.49	p<0.001
Dataset 3	Smooth motion	508.73	p<0.001
	Rough motion	551.63	p<0.001

One-way ANOVA test results for datasets 1, 2 and 3: the mean value for each category is significantly different as corroborated by the p-values. This confirms the entropy as a significant factor on the probability of the evaluation to fall in a certain category.

### 3.4 Linear regression between RMS and GE

**Fig 7:**
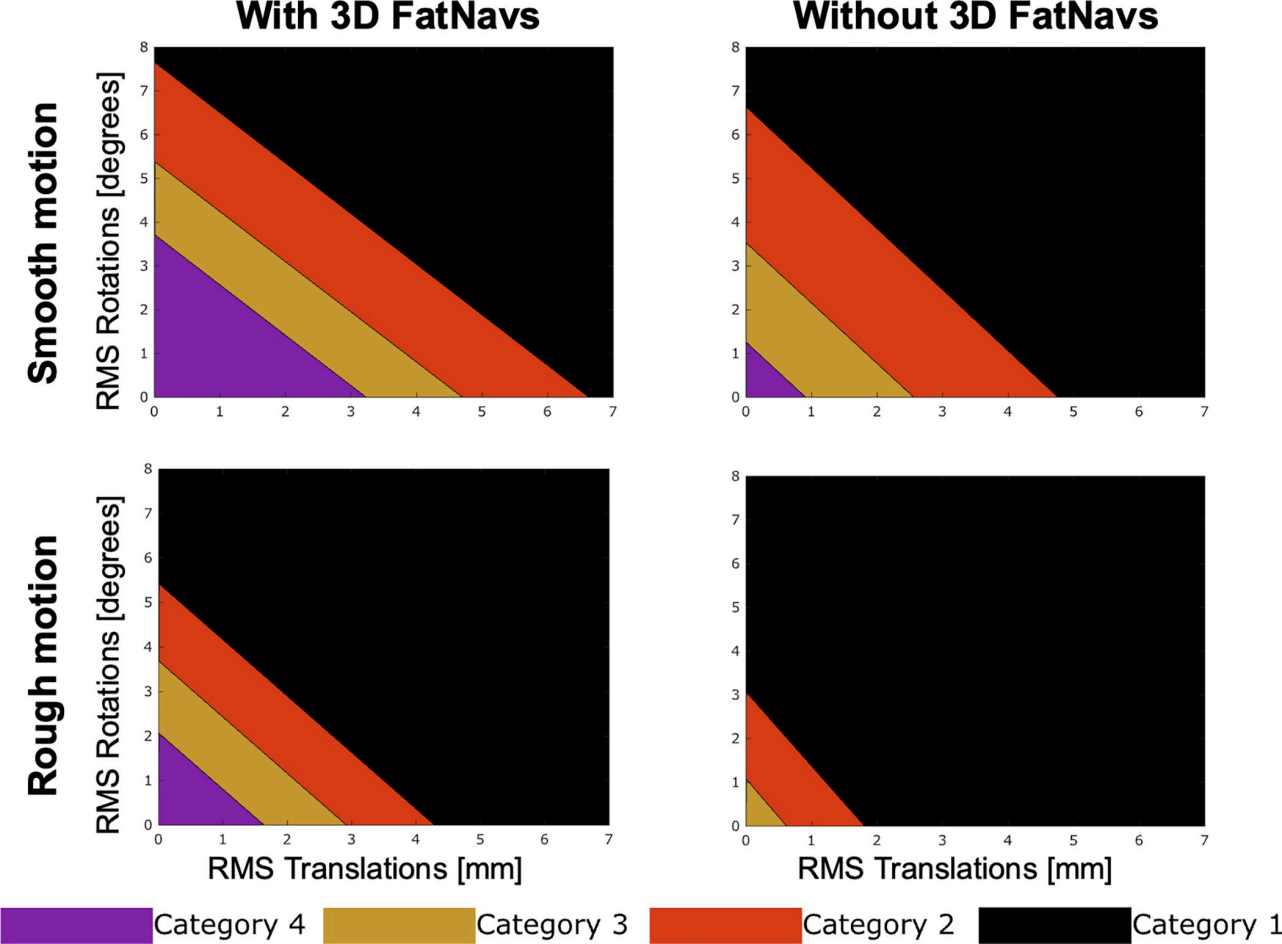
Estimated motion level at each category boundary. The motion levels were estimated as the average across the three datasets for smooth and rough motion in the cases of 3D FatNav-based correction or without motion correction. 3D FatNav-based correction shows a high tolerance to motion, whereas the image quality decreases faster for MPRAGE images without motion correction. Category definition: 4 = no visible motion artifacts, 3 = some motion artifacts, 2 = strong motion artifacts and 1 = severe motion artifacts.

It was found that the relationship between rotational, translational motion and the GE could be described by the linear model defined in Eq 1: the use of the interaction term resulted in a lower BIC score compared to the model without, confirming that the interaction term provided a better fit to the data. All the statistical parameters resulting from the linear regression test are reported in Table 3. The linear model was used to estimate the rotational and translational motion corresponding to the GE value found at categories’ intercepts via the multinomial logistic regression. Fig 7 compares the image quality achieved by using 3D FatNav-based motion correction (MPRAGE images corrupted with residual motion as described in Fig 4) against no-motion correction: each colored region bounds the rotational and translational motion parameters range for each evaluation category with 3D FatNav-based correction and without motion correction, in the cases of smooth and rough motion. 3D FatNav-based correction is shown to perform very well for an RMS value along the three axes of up to ∼3.7°/3 mm and ∼2°/1.6 mm for smooth and rough motion respectively (category 4 boundary), while motion-parameter estimation accuracy drops above ∼5.3°/4.6 mm and ∼3.7°/2.9 mm (category 2 boundary). On the other hand, image quality falls much more quickly without motion correction, where the category 2 boundary is ∼3.5°/2.5 mm for smooth motion and ∼1°/0.6 mm for rough motion. RMS values at each category boundary are reported in Table 4, for rotational and translational motion, with and without using 3D FatNav-based motion correction: in presence of rough motion, it seems not possible to achieve a category 4 image quality (no visible artifacts) without the motion-correction.

**Table 3:** Statistical parameters of the linear regression model.

Dataset	Motion	R-squared	F-value (4, 92)	p-value	BIC with interaction term	BIC w/o interaction term
Dataset 1	Smooth motion	0.77	103.29	p<0.001	-106.76	-102.19
	Rough motion	0.87	215.95	p<0.001	-168.00	-151.37
Dataset 2	Smooth motion	0.84	166.97	p<0.001	-75.32	-57.59
	Rough motion	0.80	119.34	p<0.001	-87.97	-65.44
Dataset 3	Smooth motion	0.83	152.67	p<0.001	-40.63	-32.72
	Rough motion	0.81	137.65	p<0.001	-84.90	-74.05

Linear regression model statistical parameters for each dataset in the cases of smooth and rough motion; the F-values and related p-values, as well as the R-squared values, denote that the model selected adequately explains the data both in the case of smooth and rough motion; moreover, models with the interaction term (between rotations and translations) exhibit a lower BIC value compared to models without.

**Table 4:** RMS values at image quality category boundaries.

Category	Smooth motion				Rough motion			
	With 3D FatNavs		No motion correction		With 3D FatNavs		No motion correction	
Category 4 to 3	3.18 mm	3.67°	0.87 mm	1.21°	1.55 mm	1.97°	0 mm	0°
Category 3 to 2	4.63 mm	5.32°	2.48 mm	3.44°	2.71 mm	3.43°	0.47 mm	0.78°
Category 2 to 1	6.81 mm	7.83°	4.94 mm	6.85°	4.10 mm	5.19°	1.67 mm	2.81°

Translational and rotational RMS values at each category boundary for smooth and rough motion if 3D FatNav-based motion correction was used.

### 3.5 Linear and non-linear regression between FD and GE

**Fig 8:**
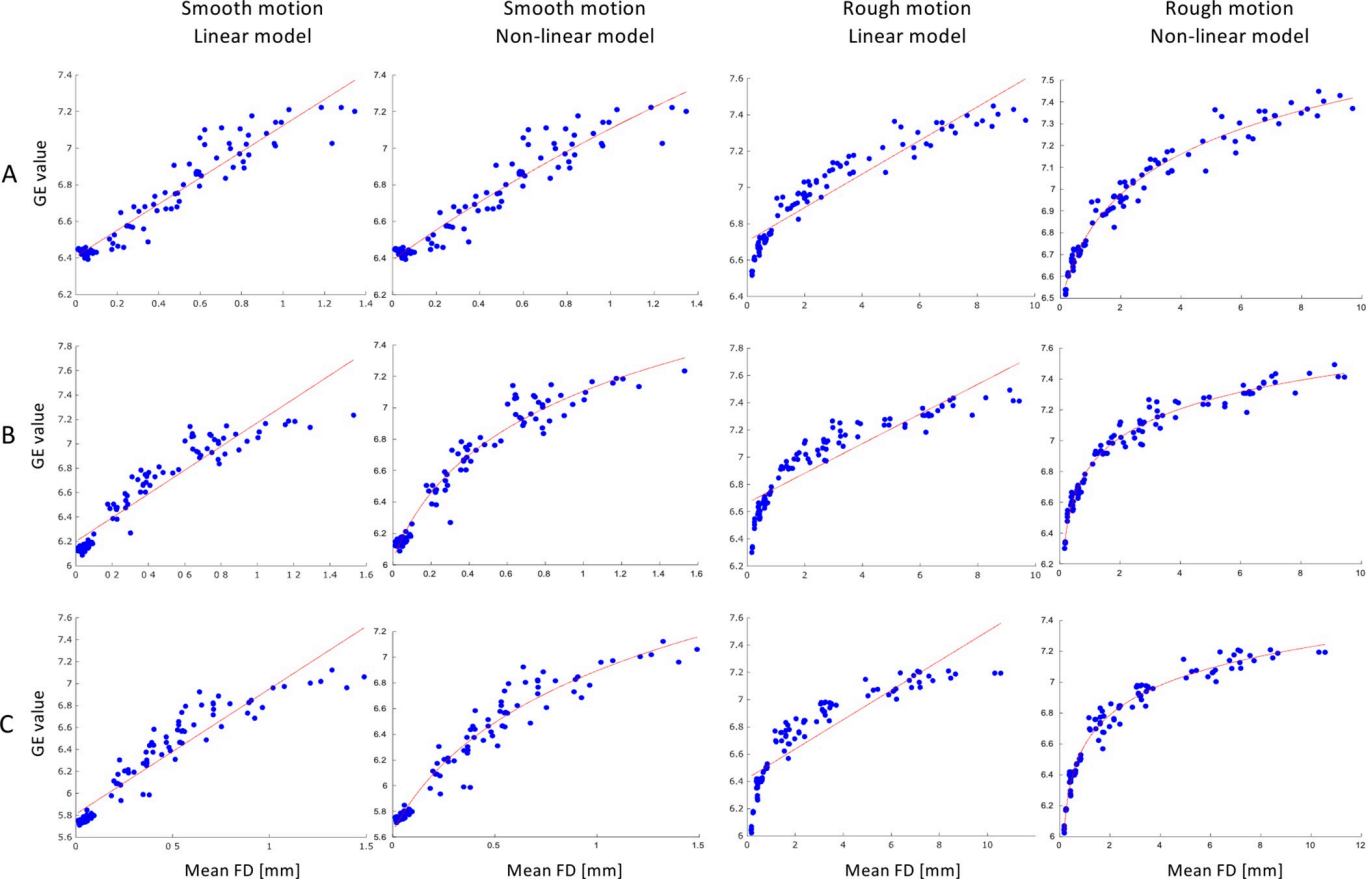
Comparison of the linear and non-linear model fitting of the GE values as a function of the mean FD in case of smooth and rough motion. The mean FD values were estimated from the residual motion resulting after 3D FatNav-based motion correction for dataset (A) 1, (B) 2 and (C) 3. The mean FD values estimated from the rough motion case are much larger (up to 12 mm for Dataset 3) compared to the FD range displayed for smooth motion. Nevertheless, the non-linear model is shown to fit the data more accurately than the linear model both for smooth and rough motion. This is corroborated by the estimated R-squared values for the three datasets: the estimated values for smooth and rough motion were 0.91/0.88/0.87 and 0.87/0.78/0.75 using the linear regression model, and 0.95/0.97/0.97 and 0.98/0.98/0.98 when using non-linear regression model, showing a clear improvement in the fitting when the latter is adopted.

The mean FD values were estimated from the residual motion resulting after applying the 3D FatNav-based motion correction (see Fig 4) in the presence of smooth and rough types of motion. The statistical relationship between the mean FD and GE was investigated, comparing a linear and a logarithmic regression model (Eq 2). The two models are compared in Fig 8: the linear regression model provided a good fit to the smooth motion data, with an R-squared value of 0.91/0.88/0.87 for the three datasets. However, the quality of the fitting decreased in the case of rough motion, with R-squared values being 0.87/0.78/0.75. On the other hand, the non-linear regression model provided an accurate fitting both in the presence of smooth and rough motion for all three datasets, with R-squared values of 0.95/0.97/0.97 and 0.98/0.98/0.98 respectively. This denoted that although the linear regression model could potentially describe well both smooth and rough motion, the data was explained considerably better by the non-linear model.

The non-linear regression model was therefore used to find the categories’ boundaries based on the mean FD for both smooth and rough motion. If 3D FatNav-based correction was used, image quality degraded to a “some motion artifacts” level (category 3) for FD values of 0.10 mm and 0.23 mm for smooth and rough motion respectively. The image quality was estimated to degrade to a level of “strong motion artifacts" (category 2) for values of 0.21 mm and 0.45 mm, while an image quality level of “severe motion artifacts” (category 1) was achieved for FD values larger than 0.44 mm and 1.13 mm for smooth and rough motion respectively.

## 4. Discussion

In this study, we assessed a simulation of the accuracy of motion parameter estimation from 3D FatNavs across a broad range of head motion. GRAPPA reconstruction of the 3D FatNav volumes was expected to perform poorly in the case of large head position changes, because of inconsistencies between the ACS lines and the 3D FatNavs volume being reconstructed. The effect of this mismatch is shown in Fig 5: the Corrupted 3D FatNav volumes image quality degraded compared to the transformed volumes as the motion increases from "low" to "very high". Two observers were asked to evaluate a total of 576 MPRAGE images from 1 (severe motion artifacts) to 4 (no visible motion artifacts) using a GUI in MATLAB. The multinomial logistic regression was performed to test the relation between the observers’ scores and the GE, and to understand when the evaluation changed based on the entropy value. Fig 6 shows that the probability of falling into category 4 (no visible motion artifacts in the MPRAGE images) decreased as the GE value increased, as expected. Nevertheless, the boundary between each category was found to be better defined in the case of rough motion compared to smooth motion: Fig 6 shows that the probability does not exceed 0.5 for category 3 and 2 in Dataset 1 and for category 3 in Dataset 3 in presence of smooth motion. However, it is very close to 1 when rough motion affects the images. This is due to the fact that the image quality degrades much more quickly in the case of rough motion compared to smooth, with fewer ambiguous cases which could be assigned to both categories.

Observers are expected to have less difficulty discerning between images with some motion artifacts (category 3) and with strong motion artifacts (category 2), producing even a stronger agreement among evaluators. Moreover, the observers were asked to evaluate the image based on its overall quality: in future studies, trained neuroradiologists would be asked to assess the quality of clinical images based on the expected ability to discern abnormality of different sizes, which is expected to lead to more accurate evaluations.

Although 3D FatNav-based correction was demonstrated to correct for a large scale of motion, the GRAPPA reconstruction of the 3D FatNav volumes themselves will be compromised in the presence of strong head position changes. This may lead to a misestimation of the motion parameters. Nonetheless, Fig 7 shows that for levels of motion (as RMS) between 3.7° and 3 mm, GRAPPA reconstruction has little effect on 3D FatNav-based motion estimation accuracy: our data suggests that motion that would be sufficient to lead to a category 2 rating (strong artifacts) can typically be corrected to a level corresponding to category 4 (no noticeable artifacts). Especially in the case of rough motion, 3D FatNav-based motion correction was proven to be indispensable, as a category 4 image quality could not be achieved without motion correction (Table 4). If a particular subject group is likely to move more than this, it may be necessary to adapt the 3D FatNavs acquisition to make it more robust to large motion, which is expected to be possible if the GRAPPA weights are dynamically updated during the scan. This step could be particularly beneficial in the case of high-resolution imaging, where the effect of GRAPPA misestimation due to head motion is expected to be more prominent.

The relationship between the mean FD and the GE was investigated by comparing how well the linear and non-linear regression models could fit the data with smooth and rough motion. It was found that the logarithmic model could fit both our rough and smooth data much more accurately compared to the linear model, as shown in Fig 8. This could be due to the entropy, used here as image quality metric, being based on the log-function. Only the first dataset affected by smooth motion reported an R-squared value slightly lower (0.95) compared to the other datasets, as shown in Fig 8A. It is possible that a different model would provide an even better description of our data, which would lead to a more accurate estimation of the FD values that define each image quality category.

**Fig 9:**
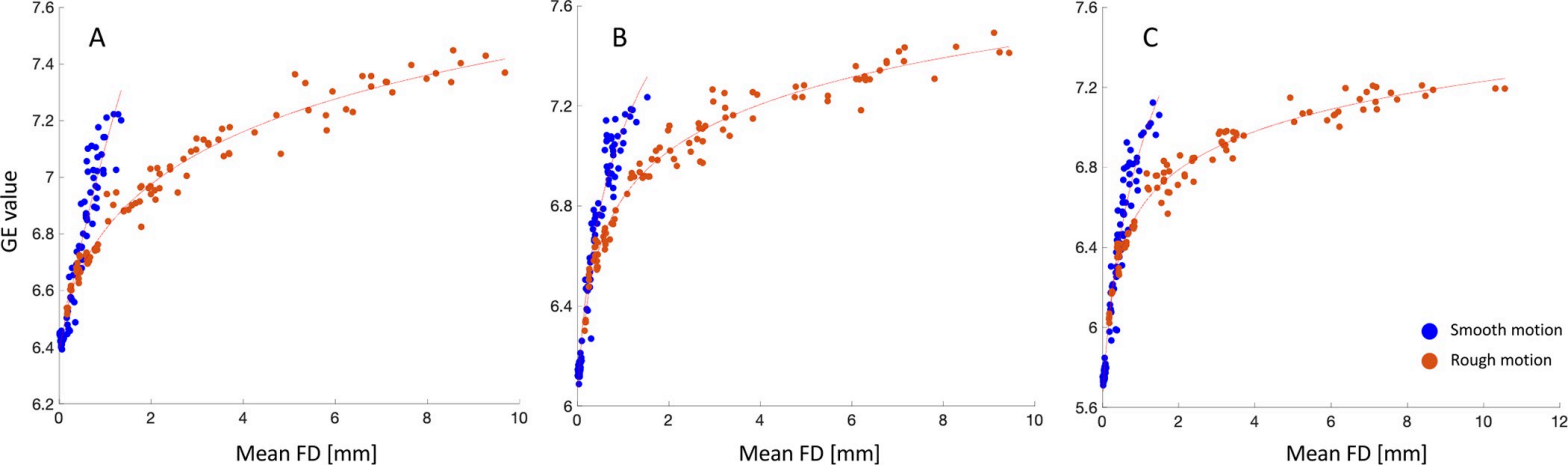
Comparison between mean FD values for smooth and rough motion parameters. The mean FD values were estimated using the residual motion (remaining motion after 3D FatNavs motion correction) in presence of smooth (blue dots) and rough (orange dots) motion for datasets (A) 1, (B) 2 and (C) 3. A logarithmic regression model (described in Fig 8) was fitted to the data and is plotted as a continuous red line. The mean FD is shown to differentiate between smooth and rough motion, except for mean FD values up to ~1.2 mm which are shown to overlay. This suggests that, if appropriately scaled, the FD metric could be adopted to measure the quantity of motion as well as to discern between types of motion which occurred during an MRI acquisition.

Unlike the RMS, which averages the motion parameters across the entire scan duration, the FD metric estimates the head position change between consecutive volumes throughout the acquisition, providing a more accurate depiction of the effects of the smooth and rough motion cases described in this work. As shown in Fig 8, our rough motion parameters resulted in much higher FD values compared to smooth motion ones for similar entropy values, with the FD increasing up to 10 mm in Datasets 1 and 2 and up to 12 mm in Dataset 3, while it did not exceed 1.6 mm in the case of smooth motion. This difference between smooth and rough motion can be further appreciated in Fig 9, suggesting that, if appropriately weighted, the FD value could then be used as a metric to measure the extent of the motion corruption affecting the MRI acquisition–a topic that will be explored in future work. Although a definition of smooth and rough motion was given, further investigation may be required to find a useful mathematical way to describe the difference between them. It is expected that most routine MRI acquisitions are affected by smooth motion, where subjects move slowly throughout the acquisition; however, rough motion is expected to be more characteristic of the motion profiles exhibited less compliant subjects during MRI scans. The clear differences shown here in artifacts between the two categories suggest that being able to distinguish between smooth and rough motion becomes important to be able to test motion correction accuracy for different types of motion.

## 5. Conclusions

In this study, data from five MPRAGE brain images acquired at 3T were used to estimate the motion corresponding to four image quality boundaries and assess motion tolerance when 3D FatNav-based motion correction is used. We showed an increase of parallel imaging artifacts on the 3D FatNav volumes for larger changes in the head position. Nonetheless, our data suggests that strong motion artifacts can be corrected with 3D FatNav-based motion-correction to a level corresponding to no noticeable artifacts, demonstrating a high tolerance to motion compared to when no motion correction is applied. If a particular subject group is likely to move more than these levels, it may be necessary to adapt the 3D FatNavs acquisition to make it more robust to large motion, which is expected to be possible if the GRAPPA weights are dynamically updated during the scan.
